# Psychometric properties of the Trauma and Distress Scale, TADS, in an adult community sample in Finland

**DOI:** 10.3402/ejpt.v7.30062

**Published:** 2016-03-30

**Authors:** Raimo K. R. Salokangas, Frauke Schultze-Lutter, Paul Patterson, Heinrich Graf von Reventlow, Markus Heinimaa, Tiina From, Sinikka Luutonen, Juha Hankala, Mika Kotimäki, Lauri Tuominen

**Affiliations:** 1 Department of Psychiatry, University of Turku, Turku, Finland; 2 University Hospital of Child and Adolescent Psychiatry and Psychotherapy, University of Bern, Bern, Switzerland; 3 Youthspace – Birmingham & Solihull Mental Health Foundation Trust, Birmingham, United Kingdom; 4 Ev. Zentrum für Beratung und Therapie am Weißen Stein, Evangelischer Regionalverband Frankfurt am Main, Frankfurt am Main, Germany; 5 Psychiatric Clinic, Turku University Central Hospital, Åbo, Finland

**Keywords:** Childhood traumatic experiences, assessment, reliability, validity, general population

## Abstract

**Background:**

There is increasing evidence that a history of childhood abuse and neglect is not uncommon among individuals who experience mental disorder and that childhood trauma experiences are associated with adult psychopathology. Although several interview and self-report instruments for retrospective trauma assessment have been developed, many focus on sexual abuse (SexAb) rather than on multiple types of trauma or adversity.

**Methods:**

Within the European Prediction of Psychosis Study, the Trauma and Distress Scale (TADS) was developed as a new self-report assessment of multiple types of childhood trauma and distressing experiences. The TADS includes 43 items and, following previous measures including the Childhood Trauma Questionnaire, focuses on five core domains: emotional neglect (EmoNeg), emotional abuse (EmoAb), physical neglect (PhyNeg), physical abuse (PhyAb), and SexAb.

This study explores the psychometric properties of the TADS (internal consistency and concurrent validity) in 692 participants drawn from the general population who completed a mailed questionnaire, including the TADS, a depression self-report and questions on help-seeking for mental health problems. Inter-method reliability was examined in a random sample of 100 responders who were reassessed in telephone interviews.

**Results:**

After minor revisions of PhyNeg and PhyAb, internal consistencies were good for TADS totals and the domain raw score sums. Intra-class coefficients for TADS total score and the five revised core domains were all good to excellent when compared to the interviewed TADS as a gold standard. In the concurrent validity analyses, the total TADS and its all core domains were significantly associated with depression and help-seeking for mental problems as proxy measures for traumatisation. In addition, robust cutoffs for the total TADS and its domains were calculated.

**Conclusions:**

Our results suggest the TADS as a valid, reliable, and clinically useful instrument for assessing retrospectively reported childhood traumatisation.

There is growing evidence that a history of childhood abuse and neglect is not uncommon among those who experience mental disorder (Iffland, Brähler, Neuner, Häuser, & Glaesmer, 2013; Read, Hammersley, & Rudegeair, 2007; Saed, Talat, & Saed, 2013; Schüssler-Fiorenza Rose, Xie, & Stineman, 2014), and several studies have indicated that childhood trauma experiences and adverse life events are associated with adult psychopathology including personality disorders, depression, anxiety, dissociative symptoms, substance abuse, suicidal behaviour, and psychosis (Briere, Hodges, & Godbout, 2010; Draijer & Langland, 1999; Ferguson & Dacey, 1997; Pine & Cohen, 2002; Salzman et al., 1993; Soloff, Lynch, & Kelly, 2002; Triffleman, Marmar, Delucchi, & Ronfeldt, 1995; Varese et al., 2012). Thus, adverse and traumatic childhood experiences are of great interest to psychiatry, and several interview and self-report instruments for their assessment have been developed (e.g., Bernstein et al., 1994; Bernstein, Ahluvalia, Pogge, & Handelsman, 1997; Bremner, Vermetten, & Mazure, 2000; Bremner, Bolus, & Mayer, 2007; Briere and Runtz, 1990; Felitti et al., 1998; Gallagher, Flye, Hurt, Stone, & Hull, 1989; Roy & Perry, 2004; Thabrew, De Sylva, & Romans, 2012). Yet, while many focus on sexual abuse (SexAb), relatively few assess multiple types of trauma or adversity, possibly because a consistent understanding and agreed definitions of differing types of trauma and their impact is still missing (Thabrew et al., 2012). A clinician-administered assessment (the Childhood Trauma Interview, Bernstein et al., 1994) resulted in the development of a self-report inventory the Childhood Trauma Questionnaire (CTQ) (Bernstein et al., 1997) that included childhood emotional, physical, and sexual abuse, and as well as emotional and physical neglect as core domains. Another self-report questionnaire, the Early Trauma Inventory–Self Report, assesses physical, emotional, and sexual abuse, as well as general traumas (Bremner et al., 2007). Emotional and physical abuse, emotional and physical neglect, and sexual abuse are generally regarded in the literature as five core childhood adversity domains (Burgermeister, 2007; Thabrew et al., 2012). A broader concept of adversity can include peer emotional abuse (EmoAb); peer bullying; witnessing violence against a parent or sibling; bereavement and other loss; parental mental illness; stigma and discrimination; and other traumatic events such as natural disasters (Kessler, Davis, & Kendler, 1997; Teicher & Parigger, 2015; Varese et al., 2012). Reliable reporting of the psychometric properties of retrospective childhood trauma measures is also frequently lacking (Burgermeister, 2007; Pietrini, Lelli, Verardi, Silvestri, & Faravelli, 2010; Roy & Perry, 2004; Thabrew et al., 2012). From self-report scales, psychometric properties of the CTQ (Bernstein et al., 1997) and the Early Trauma Inventory–Self Report (Bremner et al., 2007) have been established.

Within the European Prediction of Psychosis Study (EPOS; Klosterkötter et al., 2005), Patterson et al. (2002) developed a new self-report instrument, the Trauma And Distress Scale (TADS) to enable the assessment of a range of adverse childhood experiences in patients at clinical high risk of psychosis. Items for the TADS were initially selected from a comparison of several scales for the assessment of traumatic, adverse, and distressing childhood events or experiences including the CTQ (Bernstein et al., 1994) and the Child Abuse & Trauma Scale (Sanders & Becker-Lausen, 1995). Additional items were gathered from a review of common childhood adversity-related issues reported by clinical staff treating individuals in youth and adult mental health services in EPOS project centres. The aim was to agree a checklist of items describing core domains of childhood adversity, and for the scale to be feasible in both self-report and interview formats for working with high-risk clinical samples and additional comparative populations. To ensure adequate content validity and psychometric consistency (Michel, Pace, Edun, Sawhney, & Thomas, 2014; Streiner, 1993), frequency ratings employing a five-point Likert-scale focused on the five core domains: emotional neglect (EmoNeg) and emotional abuse (EmoAb), physical neglect by parents/caregivers (PhyNeg), physical abuse (PhyAb), and sexual abuse (SexAb) by non-specified offenders. Other items assess loss events, discrimination, bullying, and guilt, and two items represent a “lie scale.”

To examine other important psychometric properties of the TADS (Michel et al., 2014; Streiner, 1993), we examined (1) inter-method reliability between the self-rated and interviewed TADS and (2) internal consistency of the five TADS trauma domain sub-scales as a measure of reliability. In addition, employing “level of depression” and “help-seeking for mental health difficulties” as proxy measures for traumatisation in the broadest sense, we examined the concurrent validity of the TADS and developed domain-specific cutoffs, whilst also considering the impact of potentially confounding conditions such as age, gender, and education.

## Methods

The ethical committee of the University of Turku and the Turku University Central Hospital approved the study protocol.

### Sample

A random, age stratified sample of 2,080 citizens aged 18 years or more was drawn from the general population of the Varsinais-Suomi Health District of South-West Finland. The general sampling rate was 1/100, and, because of their low proportion in the population, 2/100 for people over 70 years. An extensive questionnaire battery was mailed in spring 2008 and re-mailed to non-responders in summer 2008. In the first round 545 (26.2%) and in the second round 147 (7.1%) subjects responded, thus one-third (*N*=692, 33.3%) of the sample returned the completed questionnaire. Response rates for females (41.5%) were higher than that for males (25.3%; Fisher exact: *p*<0.001). Mean age of responders (42.0±16.95 years) was slightly higher than that of non-responders (39.5±16.37 years; *p*=0.001).

In addition, a random sample of 100 responders were contacted, and items from the TADS were reassessed in a semi-structured telephone interview. The interviewers, three medical students, were blind to the questionnaire responses from the earlier completed TADS. The time period from return of the completed questionnaire to interview ranged from 2 to 4 weeks.

### Assessments

The questionnaire battery included items on participants’ socio-demographic background and prior help-seeking for mental health problems from a psychiatric service as well as the TADS. Originally, the TADS was developed in English (Birmingham). Three other EPOS centres (Cologne, Amsterdam and Turku) translated it into their own native language. In Turku, the TADS was translated in Finnish by one of our study group (MH) and back-translated by a professional translator of English. Since the TADS was subsequently available in Finnish and as there was no other existing Finnish scale fit for the purpose of measuring childhood adversity, several research groups began to use it in various populations. Initially, we have selected a general population for the assessment of the TADS's basic psychometric properties. We have also planned to evaluate its properties in clinical samples.

The TADS includes 43 items (Supplementary file and Table 1) on childhood trauma and adversity rated for their frequency in a Likert format: 0=“never,” 1=“rarely,” 2=“sometimes,” 3=“often,” and 4=“almost always.” To control for possible response bias, questions were phrased both positively (high ratings indicative of adversity) and negatively (low ratings indicative of adversity). Thus ratings of negatively phrased items (r) require reversion before calculation of the total and five domain scores. Two items (18 and 27) rating exaggerated positive responses operated as a “lie scale” for the purposes of validity. Five TADS domain scores can be calculated by summing their five respective items: (1) EmoNeg (5r, 8r, 13r, 21r, 40r), (2) EmoAb (10, 12, 14, 26, 32), (3) PhyNeg (1r, 2, 4, 6, 31r), (4) PhyAb (9, 16, 17, 20, 24), and (5) SexAb (22, 25, 30, 33, 41) as well as the TADS total trauma score (sum of all five domain scores). In descriptive statistics, we also calculated the TADS total score for all 43 items. Proportion of missing data on individual items of the TADS was generally lower than 1% except for the PhyAb items 17 (2.6%) and 20 (1.0%), the EmoNeg item 21 (1.2%), and items 29 (1.9%; feeling singled-out) and 38 (1.0%; loss event).

The questionnaire battery also included the depression screening instrument DEPS (Salokangas, Poutanen, & Stengård, 1995) consisting of 10 questions rated on a Likert scale as: 0=“not at all,” 1=“to some extent,” 2=“rather much,” and 3=“very much”); their sum indicates number of depressive symptoms during the past month. In a sample of patients attending primary care (Salokangas et al., 1995) at a cutoff of >8, the DEPS revealed a sensitivity of 74% and a specificity 85% for clinical depression.

Data on previous psychiatric treatment (help-seeking) and DEPS was available from all but three of the 692 subjects.

### Statistical analyses

Data were analysed using Statistical Programme for the Social Sciences (SPSS) v22.0. To calculate the inter-method reliability between self-report and interview, intra-class coefficients (ICC) were calculated for the raw score of each TADS item. In addition, each TADS item was dichotomised [0=0 (“never”) to 1 (“rarely”), and 1=2 (“sometimes”) to 4 (“almost always”)], reversed for negatively phrased items. Agreement for presence of adverse childhood experiences across questionnaire and interview was calculated by the overall concordance rate (CR) and additionally by Cohen's kappa (κ) statistic to control for effect of chance. ICC values of less than 0.40 indicate poor, 0.40–0.59 fair, 0.60–0.74 good, and 0.75–1.0 excellent agreement (Cicchetti, 1994). According to Burn, Pitchard, and Whay (2009), κ≥0.40 and CR≥75% are considered clinically useful. A disadvantage generally associated with use of κ is its dependence on the prevalence of an event (Byrt, Bishop, & Carlin, 1993); κ tends to decrease when a response/event is rare, even if the CR is high. In the absence of a satisfactory mathematical solution to this problem, we followed the approach for the appraisal of κ suggested by Burn and Weir (2011) and additionally calculated the prevalence index (PI) when information was contradictory, that is, when CR exceeded 75% but κ fell below 0.40. The PI reports values between −1 and 1, and is 0 when both responses are equally probable (i.e., their prevalence is 50%). With PI→∣1∣, the likelihood of an underestimation of κ increases, and more attention should be paid to CR.

With regard to the five core domains, both raw (range 0–20) and dichotomised (range 0–5) scores of their respective items were summed as a measure of severity of trauma and adversity in each domain, and ICCs were calculated. Following this, the domain severity scores were again dichotomised (0=0; 1=1–5) as an indicator of persons (“cases”) who rated ≥2 (“sometimes”) in ≥1 items of the respective domain and, thus, had suffered from some childhood adversity in this respect. To calculate the inter-method reliability of this binary score, CR, κ, and PI were calculated again.

To examine the internal consistency of domains, Cronbach's alphas (α) were calculated for sum scores of both original raw items and dichotomised items of the domain. For the evaluation of α the following rules were applied: >0.90=excellent, 0.80–0.89=good, 0.70–0.79=acceptable, 0.60–0.69=questionable, and ≤0.59=poor (George & Mallery, 2003).

Using current depression (DEPS>8) and help-seeking for mental problems as proxy measures of adverse experiences, we examined the concurrent validity of the TADS by cross-tabulating each of these two proxies with TADS domain “cases,” and diagnostic accuracy measures (sensitivity, specificity, positive and negative predictive values, positive and negative likelihood ratios (LRs)) were then calculated for TADS domain “cases.” LRs can guide the estimation of concurrent validity for the availability of interpretation guidelines (Jaeschke, Guyatt, & Sackett, 1994) that are missing for other accuracy measures that can only be interpreted by less reliable rules-of-thumb (Boyko, 1994; Jaeschke et al., 1994).

## Results

### Distribution and frequency of items and core domains

Frequencies of individual items are shown in Table 1 and descriptive statistics for TADS domain scores in Table 2.

**Table 1:** TADS items: original (i.e., unrevised) score frequencies (in %), proportion of item scores ≥2 in the general population sample (*N*=692), and inter-method reliability of self-rating of “≥2” to gold-standard interview assessment (*N*=100)

Item-nr.	Statement	0 never	1 rarely	2 sometimes	3 often	4 nearly always	ICC (raw score)	≥2	CR for “≥2”	κ for “≥2”	PI^a^ for “≥2”
1(r)^3^	When I was young, I felt safe and protected by someone	1.9	5.6	10.8	27.6	54.0	0.809	18.4	0.880	0.639	
2^3^	When I was young, I was often hungry	44.1	31.8	15.2	7.1	1.9	0.538	24.1	0.790	0.364	−0.090
3	I was bullied at school	28.9	36.1	24.4	7.1	3.5	0.802	35.0	0.750	0.457	
4^3^	I often wear ragged or dirty clothes to school	71.4	16.2	7.4	4.0	1.0	0.841	12.4	0.920	0.717	
5(r)^1^	When I was young, I felt valued or important	3.9	9.8	17.5	33.8	35.0	0.829	31.2	0.840	0.620	
6^3^	My parents/caregivers were often drunk, stoned, or wasted	64.0	18.8	8.1	8.4	0.7	0.922	17.2	0.940	0.797	
7	I have been bullied at work	65.3	23.6	8.1	2.3	0.7	0.918	11.1	0.910	0.657	
8(r)^1^	My family was emotionally warm and loving	3.8	7.7	17.6	27.2	43.8	0.852	29.0	0.850	0.619	
9^4^	When I was young, I was hit so hard that it left marks, cuts, or bruises	82.1	9.8	4.6	2.9	0.6	0.865	8.1	0.960	0.811	
10^2^	I felt rejected by my parents/caregivers	66.5	15.5	10.4	5.6	2.0	0.804	18.1	0.880	0.611	
11(r)	When I was young, there was an adult I could confide in	7.2	11.4	9.0	22.9	49.5	0.803	27.6	0.840	0.574	
12^2^	When I was young, I was humiliated by people in my family	63.2	17.3	9.4	6.1	4.0	0.831	19.5	0.850	0.615	
13(r)^1^	When I was young, my family looked after each other	2.7	6.6	13.3	28.5	48.8	0.809	22.7	0.900	0.688	
14^2^	I believe that I am a bad person	62.9	26.2	8.2	1.6	1.2	0.793	11.0	0.930	0.593	
15	I believe that somebody died because of me	93.1	3.0	1.3	1.2	1.4	0.835	3.9	0.980	0.740	
16^4^	I have experienced serious physical assault	82.8	9.5	5.6	1.6	0.4	0.709	7.7	0.920	0.592	
17^4^	Adults noticed cuts, bruises, or marks from when I was beaten	94.9	2.6	1.4	0.3	0.7	0.084	2.5	0.950	0.025	0.010
18(r)	My childhood was perfect	8.5	9.4	25.1	39.5	17.5	0.840	43.1	0.800	0.584	
19	I am bothered by a very shameful secret	74.6	14.2	6.5	3.2	1.5	0.865	11.1	0.910	0.589	
20^4^	I think I was physically abused when I was young	83.4	7.7	4.8	3.2	1.0	0.865	9.0	0.920	0.670	
21(r)^1^	I respect myself	2.2	4.0	16.8	36.3	40.8	0.634	23.0	0.870	0.457	
22^5^	When I was young, someone touched me or tried to make me touch them in a sexual way	89.0	6.8	3.0	1.0	0.1	0.795	4.2	0.960	0.646	
23	I have had experiences that I feel very guilty about	46.7	36.4	12.1	3.9	0.9	0.635	16.9	0.840	0.243	0.020
24^4^	I have been involved in life-threatening situations	64.7	20.8	12.0	2.5	0.0	0.788	14.5	0.850	0.542	
25^5^	I was forced to keep secrets about someone sexually interfering with me when I was young	97.0	1.4	0.9	0.1	0.6	0.902	1.6	0.980	0.658	
26^2^	When I was young, I felt hated by a member or members of my family	77.2	11.1	6.6	4.0	1.0	0.893	11.7	0.940	0.765	
27(r)	My family was the greatest ever	8.7	8.8	17.2	31.1	34.2	0.751	34.7	0.820	0.570	
28	Other people have acted badly because of me	64.6	26.6	7.7	1.2	0.0	0.683	8.8	0.930	0.551	
29	When I was young, I felt like the odd one out in my family	58.7	19.8	13.2	4.8	3.6	0.873	21.5	0.890	0.724	
30^5^	I have experienced sexual assault	95.8	2.3	1.4	0.3	0.1	0.815	1.9	0.990	0.795	
31(r)^3^	If I needed treatment someone would always take me to see a doctor or nurse when I was young	3.8	4.5	7.2	16.5	68.1	0.671	15.5	0.880	0.557	−0.020
32^2^	I feel that I was put down, criticised, and made to feel inferior when I was young	56.8	19.4	10.0	10.8	3.0	0.863	23.8	0.860	0.645	
33^5^	Someone sexually molested me when I was young	91.8	5.8	2.0	0.4	0.0	0.766	2.5	0.970	0.559	
34	I feel responsible for harm and injury to another person	82.5	11.4	3.9	1.3	0.9	0.609	6.1	0.920	0.386	−0.020
35(r)	When I was young, I had friends I could talk to about personal problems	10.1	13.7	14.6	31.9	29.6	0.763	38.4	0.800	0.576	
36	I have experienced harassment/persecution from other ethnic groups	97.0	1.3	1.2	0.4	0.1	0.382	1.7	0.990	0.000	−0.010
37(r)	I did well at school	1.4	6.9	31.2	33.2	27.2	0.742	39.6	0.780	0.490	
38	I have experienced the loss of somebody who was very important to me	26.6	29.5	26.7	7.8	9.4	0.615	43.9	0.660	0.323	−0.120
39	I believe that I do not deserve to do well in life	67.0	20.3	9.7	2.5	0.6	0.623	12.7	0.890	0.304	−0.070
40(r)^1^	My family was supportive and encouraging when I was young	6.1	9.8	16.9	24.9	42.3	0.834	32.8	0.820	0.589	
41^5^	I believe that I was sexually used when I was young	94.5	2.7	1.9	0.7	0.1	0.936	2.7	1.000	1.000	
42	I felt afraid of someone in my family	59.0	15.8	12.3	6.6	6.4	0.860	25.3	0.810	0.541	
43(r)	When I was young I could make friends easily	3.2	9.8	21.0	36.8	29.2	0.835	34.0	0.850	0.590	

a The prevalence index (PI) was only calculated if for Cohen's κ and the CR produced contradictory results according to their guidelines for clinical usefulness (Burn et al., 2009), that is, κ≥0.40 and CR≥75%.

(r) indicates items whose score was revised prior to creating the binary “≥2” score or calculating sum scores and intra-class coefficient (ICC).

1 item of EmoNeg

2 item of EmoAb

3 item of PhyNeg

4 item of PhyAb

5 item of SexAb.

**Table 2:** TADS core domains: descriptive statistics and internal consistency by Cronbach's α (*N*=692), as well as construct validity of totals by the intra-class coefficient (ICC) and of at least any one domain item ≥2 by CR, κ, and PI (*N*=100)

	TADS domains	Mdn	Mean	SD	Range	≥2^a^ (%)	*α*^b^	*α*^c^	ICC^b^	self-rating^a^ (%)	interview^a^ (%)	ICC^c^	CR	κ	PI
EmoNeg	Emotional neglect (max. 20)	4.00	4.88	4.36	0–19	51.2	0.874	0.831	0.915	51.0	40.0	0.899	0.790	0.543	−0.090
EmoAb	Emotional abuse (max. 20)	2.00	3.08	4.00	0–19	37.4	0.859	0.794	0.928	40.0	41.0	0.908	0.830	0.647	−0.190
PhyNeg^d^	Physical neglect (max. 20/16)	2.00	3.29	3.07	0–17	49.7	0.624	0.579	0.922	41.0	38.0	0.910	0.850	0.686	−0.210
PhyAb^d^	Physical abuse (max. 20)	0.00	1.50	2.53	0–16	23.1	0.764	0.696	0.906	36.0	38.0	0.885	0.800	0.571	−0.260
SexAb	Sexual abuse (max. 20)	0.00	0.50	1.76	0–16	5.5	0.885	0.849	0.875	7.0	9.0	0.937	0.960	0.729	−0.840
Total 1^d^	Sum score of the 5 core domains (max. 100/96)	9.00	13.26	12.66	0–81	72.3	0.918	0.894	0.958	66.0	62.0	0.893	0.740	0.437	0.280
Total 2	Sum score of the 43-item scale (max. 172)	22.00	28.47	21.37	0–123	93.6	0.940	0.920	0.956	95.0	92.0	0.943	0.910	0.262	0.870

a Proportion of subjects with any item score ≥2 in respective domain.

b Based on sum of original raw items.

c Based on sum of dichotomised “≥2” items.

d The original domains PhyNeg and PhyAb were used in descriptive statistics and internal consistency (Cronbach's α), while PhyNegR and PhyAbR were used in construct validity (ICC, CR, κ and PI and related % in self- and interview-rating).

Over 70% of the general population subjects reported that they had experienced abuse or neglect at least sometimes (Table 2) with approximately 50% of the sample reporting emotional and physical neglect with the median score for EmoNeg (4) being twice as high as that for PhyNeg (2). Abuse was less frequent, with over 37% reporting EmoAb and 23% PhyAb at a level of “sometimes” or more frequently (Table 2). 5.5% reported experience of SexAb (Table 2), mostly by indicating that they were touched or had to touch someone else in a sexual way in their childhood (item 22: 4.1%, Table 1). The least frequent item with 1.6% endorsement of “sometimes” or more was from SexAb (item 25) referring to being forced as a child to keep SexAb a secret.

### Internal consistency of the TADS and its five core domains

Internal consistency of the total TADS score of the five domains was 0.92 for sum of original raw items and 0.89 for sum of dichotomised items. Corresponding figures of the total TADS sum score of all 43 items were 0.94 and 0.92. Internal consistencies of the five domains, indicated by Cronbach's α and calculated for original raw items and for dichotomised items, were generally better for original raw items (Table 2). While internal consistency was good for EmoNeg, EmoAb, and SexAb, and acceptable for PhyAb, it was questionable for PhyNeg. When the two items with poor inter-method reliability of raw scores (Table 1) were excluded from PhyNeg (item 2) and PhyAb (item 17), respectively, internal consistency improved to 0.64 and 0.78 for original raw items and 0.60 and 0.72 for dichotomised items, respectively. When item 17 was replaced by item 42 (I was afraid of someone in my family), internal consistency of PhyAb was acceptable with Cronbach's *α*=0.79 for raw items and 0.73 for dichotomised items. Consequently, in further analyses of inter-method reliability and concurrent validity as well as normative data, the revised domains were used, that is, PhyNegR without item 2 and PhyAbR including item 42 instead of item 17.

### Inter-method reliability of items and core domains

**Fig. 1:**
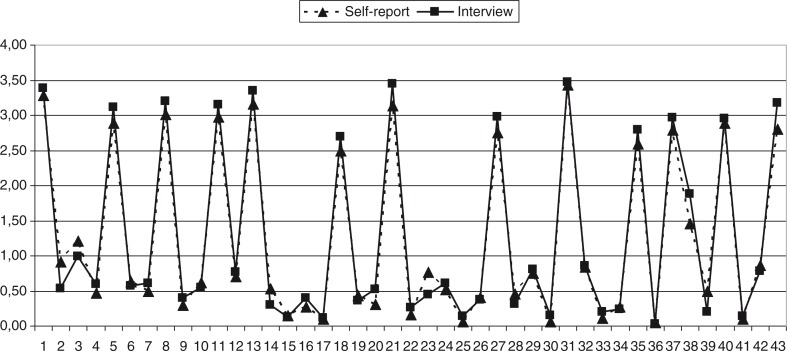
Mean scores of TADS items by self-report and interview.

As illustrated in Fig. 1, the means scores of self-reported and interview-assessed original TADS items were almost identical (Fig. 1). In line with this, inter-method reliability values of items in terms of both raw (ICC) and dichotomised scores (CR and κ) were good to excellent, the only exceptions being items 2, 17, and 36 (Table 1). For item 2 (When I was young, I was often hungry), both the ICC (0.54) for the raw score and κ (0.36) for the dichotomised score were below acceptable levels. In addition, as the PI was −0.090, the CR of 79% cannot be regarded as providing a better estimate of the inter-method reliability of the binary score; consequently item 2 has to be regarded as having insufficient inter-method reliability. The same must be assumed for item 17 (Adults noticed cuts, bruises or marks from when I was beaten), and for item 36 (I have experienced harassment/persecution from other ethnic groups); both showed excellent CRs but only moderate ICCs and κs that had to be given priority in light of low PIs (Table 1). With regard to items 23, 31, 34, 38, and 39, good to excellent ICCs of raw scores indicated that these possess better inter-method reliability than their dichotomised version where κs were insufficient at low PIs (Table 1). Compared to the interview, the self-rating of raw scores tended to give an overestimation in the case of items 2, 23, and 39 and an underestimation only for item 38, while no clear tendency could be detected for items 17, 31, 34, and 36 (Fig. 1).

As regards the five revised core domains, ICCs of totals of both raw and dichotomised scores were all good to excellent (Table 2). Furthermore, all five domains appeared to hold some clinical utility for indicating the presence of any respective adversity when compared alongside the gold standard of an interview assessment (Table 2). This did not hold for either TADS totals (Table 2); however, where by comparison the presence of any adversity was overestimated.

### Concurrent validity of the TADS and its core domains

To study the criterion validity in terms of the concurrent validity, we used presence of depression (DEPS score >8) and help-seeking from mental health services, respectively, as proxy measures of traumatisation in terms of a negative impact on mental health. In total 135 (19.6%) subjects scored >8 in the DEPS and 187 (27.1%) had sought help from a mental health service at some time in their life. For participants who had affirmed at least some experience of childhood adversity in the TADS domains and totals, depression and help-seeking were significantly more frequent at effect sizes of 0.18–0.33 and 0.14–0.30, respectively (Table 3), thus indicating good criterion validity of the TADS and all of its domains. Consistently, the effect of EmoNeg and EmoAb on the proxy measures “current depression” and “help-seeking,” were strongest, while the effect of PhyNeg was weakest (Table 3).

**Table 3:** Relationship between reported adversity, depression, and help-seeking

	Depressed (*n*=135) *n* (% of depressed)	Not depressed (*n*=554) *n* (% of not depressed)	χ^2^ (df=1), *p*; Cramer's V	Help-seeking (*n*=187) *n* (% of help-seeking)	Not help-seeking (*n*=502) *n* (% of not help-seeking)	χ^2^ (df=1), *p*; Cramer's V
EmoNeg^a^ (353 cases, 51.2%)	114 (84.4%)	239 (43.1%)	74.118, <0.001; 0.328	139 (74.3%)	215 (42.8%)	54.127, <0.001; 0.280
EmoAb (258 cases, 37.4%)	91 (67.4%)	167 (30.1%)	64.346, <0.001; 0.306	115 (61.5%)	143 (28.5%)	63.386, <0.001; 0.303
PhyNegR^b^ (342 cases, 49.6%)	91 (67.4%)	251 (45.3%)	21.209, <0.001; 0.175	115 (61.5%)	228 (45.5%)	14.090, <0.001; 0.143
PhyAbR (159 cases, 23.1%)	60 (44.4%)	99 (17.9%)	43.183, <0.001; 0.250	73 (39.0%)	86 (17.1%)	36.831, <0.001; 0.231
SexAb (38 cases, 5.5%)	21 (15.5%)	17 (3.1%)	32.480, <0.001; 0.217	24 (12.8%)	14 (2.8%)	26.384, <0.001; 0.196
TADS total of domains^c^ (498 cases, 72.3%)	126 (93.3%)	372 (67.2%)	37.146, <0.001; 0.232	163 (87.2%)	336 (66.9%)	27.929, <0.001; 0.201
TADS total (645 cases, 93.6%)	132 (97.8%)	513 (92.6%)	4.869, 0.027; 0.084	186 (99.5%)	459 (91.4%)	14.699, <0.001; 0.146

Cramer's V: 0.1=small effect; 0.3=moderate effect; 0.5=large effect.

Cases for help-seeking cross-tabulation:

a EmoNeg 354, 51.4%

b PhyNegR 343, 49.8%

c TADS total of domains 499, 72.4%.

### Normative data

With regards to potential confounders of normative data, that is, age, gender and years of education, differential effects on the TADS domains or total caseness were detected for gender and education, while effects of age were unsystematic and did not allow examination of cutoff markers (Table 4). An effect of gender was detected for EmoAb and SexAb in favour of men who reported lower presence of any adversity in these domains as indicated by standardised residuals (SR) below −1.96, while EmoNeg, PhyNegR, and PhyAbR as well as the TADS domain total were negatively associated with years of education (Table 4). Marital status, likely confounded by other variables and thus not considered separately for normative data, suggested evidence of a greater likelihood of any kind of abuse in separated, divorced, or widowed participants (Table 4).

**Table 4:** Association of socio-demographic parameters with TADS caseness, that is, scoring ≥2 in any one domain item

	n	%	EmoNeg % cases (SR)	EmoAb % cases (SR)	PhyNegR % cases (SR)	PhyAbR % cases (SR)	SexAb % cases (SR)	Total domains % cases (SR)	TADS total % cases (SR)
Gender	692								
Men	260	37.6	52.7 (0.35)	29.6 (−2.06)	50.8 (0.24)	27.7 (1.53)	1.9 (−2.46)	75.4 (0.59)	95.8 (0.35)
Women	432	62.4	50.2 (−0.27)	42.1 (1.60)	49.1 (−0.19)	20.4 (−1.19)	7.6 (1.90)	70.4 (−0.46)	92.4 (−0.28)
χ^2^, df=1, *p*			0.393, ns	10.854, ***	0.187, ns	0.174, ns	10.218, ***	2.036, ns	3.166, ns
Age	692								
18–24	81	11.7	56.8 (0.71)	44.4 (1.03)	48.1 (−0.20)	18.5 (−0.86)	3.7 (−0.69)	79.0 (0.72)	93.8 (0.02)
25–34	226	32.7	41.6 (−2.01)	25.7 (−2.89)	42.0 (−1.64)	15.5 (−2.39)	3.1 (−1.54)	61.5 (−1.90)	88.5 (−0.80)
35–44	110	15.9	46.4 (−0.70)	36.4 (−0.18)	52.7 (0.45)	23.6 (0.11)	7.3 (0.80)	72.7 (0.06)	95.5 (0.20)
45–54	86	12.4	65.1 (1.81)	48.8 (1.73)	57.0 (0.96)	34.9 (2.27)	9.3 (1.51)	80.2 (0.87)	97.7 (0.39)
55–64	87	12.6	57.5 (0.82)	43.7 (0.95)	49.4 (0.04)	26.4 (0.64)	9.2 (1.47)	74.7 (0.27)	97.7 (0.39)
65+	102	14.7	55.9 (0.67)	44.1 (1.10)	58.8 (1.31)	30.4 (1.53)	3.9 (−0.68)	81.4 (1.08)	96.1 (0.25)
χ^2^, df=5, *p*			19.320, *	23.292, ***	11.013, ns	18.658, *	8.858, ns	22.108, ***	16.438, **
Years of education	688								
≤11	168	24.4	63.7 (2.24)	44.6 (1.51)	63.7 (2.54)	35.7 (3.45)	8.3 (1.65)	85.7 (2.03)	98.2 (0.62)
12–15	299	43.5	52.8 (0.37)	36.5 (−0.30)	48.5 (−0.33)	21.4 (−0.56)	5.7 (0.23)	70.2 (−0.44)	93.3 (−0.05)
≥16	221	32.1	39.8 (−2.38)	33.5 (−0.97)	41.2 (−1.83)	15.4 (−2.35)	2.7 (−1.71)	65.2 (−1.26)	90.5 (−0.48)
χ^2^, df=2, *p*			22.269, ***	5.317, ns	19.743, ***	23.025, ***	6.019, ns	21.398, ***	9.570, **
Marital status	688								
Single	136	19.8	55.1 (0.67)	41.2 (0.73)	41.2 (−1.39)	19.9 (−0.76)	5.1 (−0.19)	70.6 (−0.23)	93.4 (−0.03)
Married	289	42.0	47.1 (−0.94)	32.9 (−1.25)	50.9 (0.31)	20.1 (−1.03)	3.5 (−1.49)	69.6 (−0.54)	93.8 (0.03)
Cohabiting	169	24.6	47.9 (−0.58)	32.0 (−1.15)	51.5 (0.35)	18.9 (−1.09)	6.5 (0.55)	71.0 (−0.19)	91.7 (−0.25)
Separated/divorced/widowed	94	13.7	62.8 (1.59)	55.3 (2.85)	54.3 (0.65)	43.6 (4.18)	10.6 (2.11)	85.1 (1.47)	96.8 (0.32)
χ^2^, df=3, *p*			8.577, ns	18.402, ***	5.099, ns	26.328, ***	7.422, ns	9.115, ns	2.644, ns

ns *p*≥0.05

* *p*<0.05

** *p*<0.01

*** *p*<0.001 in *χ*^2^-test; for each parameter adjusted for multiple testing across the six TADS totals.

SR: standardised residuals; values >∣1.96∣ indicate significant cell number deviations at *p*>0.05.

In general, diagnostic accuracy measures of binary TADS caseness gave comparable figures for both proxy measures (Table 5). As expected, sensitivity for total of TADS domains and of total TADS scale were very high, but specificity low, especially for the total scale. Totals for TADS domains demonstrated high sensitivity but lower specificity to depressiveness. From the TADS domains, SexAb showed low sensitivity but high specificity for both depressiveness and help-seeking and moderate positive LR for depressiveness. Also PhyAbR showed quite low sensitivity but high specificity. For other TADS domains, sensitivity and specificity figures were relatively balanced.

**Table 5:** Diagnostic accuracy of TADS caseness for depressiveness and help-seeking, respectively, as proxy measure of traumatisation

	Sensitivity depression	Specificity depression	PPV depression	NPV depression	PLR depression	NLR depression
TADS caseness (at least any one item of ≥2)						
EmoNeg	0.844	0.569	0.323	0.938	1.958	0.274
EmoAb	0.674	0.699	0.353	0.898	2.239	0.466
PhyNegR	0.674	0.547	0.266	0.873	1.488	0.596
PhyAbR	0.444	0.821	0.377	0.858	2.487	0.676
SexAb	0.156	0.969	0.553	0.825	5.032	0.871
Total of domains	0.933	0.329	0.253	0.953	1.390	0.204
Total of scale	0.978	0.074	0.205	0.932	1.056	0.300
	Sensitivity help-seeking	Specificity help-seeking	PPV help-seeking	NPV help-seeking	PLR help-seeking	NLR help-seeking

TADS caseness (at least any one item of ≥2)						
EmoNeg	0.743	0.572	0.393	0.857	1.736	0.449
EmoAb	0.615	0.715	0.446	0.833	2.159	0.538
PhyNegR	0.615	0.546	0.335	0.792	1.354	0.705
PhyAbR	0.390	0.829	0.459	0.785	2.279	0.736
SexAb	0.257	0.902	0.495	0.765	2.630	0.824
Total of domains	0.658	0.699	0.449	0.846	2.187	0.489
Total of scale	0.995	0.086	0.288	0.977	1.088	0.062

PPV=positive predictive value, NPV=negative predictive value, PLR=positive likelihood ratio (guidance for interpretation of the increase in the likelihood of event: >10=large and often conclusive; 5–10=moderate; 2–5=small; 1–2=minimal; 1=none), NLR=negative likelihood ratio (guidance for interpretation of the decrease in the likelihood of event: ≥0.5=minimal; 0.2–0.5=small; 0.1–0.2=moderate; <0.1=large and often conclusive).

In total, 102 (14.7%) subjects scored a maximum “4” on the “lie scale” items 18 and 27. However, Spearman's correlations between depression and TADS domains were very similar when the total sample (*N*=689) and the subsample without positive “lie scale” subjects (*N*=588) were compared: EmoAb: 0.438 versus 0.420, PhyAb: 0.262 versus 0.239, SexAb: 0.205 versus 0.190, EmoNeg: 0.431 versus 0.402, PhyNeg: 0.273 versus 0.248, TADS total: 0.451 versus 0.423. Therefore subjects who scored “4” in both items 18 and 27 were not excluded from analyses.

## Discussion

Within the EPOS project, the TADS was developed as a brief self-report scale of childhood adversity and trauma covering several core domains as well as tapping into other aspects of a broad concept of adversity (Thabrew et al., 2012). Employing a large general population sample, the current study examined major psychometric properties of the TADS and possible normative data which is often lacking for similar measures (Burgermeister, 2007; Pietrini et al., 2010; Thabrew et al., 2012).

### Reliability: internal consistency

With the exception of the PhyNeg sub-scale that displayed only borderline internal validity even after revision, all other trauma subscales exhibited acceptable or excellent internal consistency indicating that the TADS and its subscales reliably assess the target construct of retrospective “childhood trauma.”

### Inter-method reliability

Inter-method reliability as measured between self-reported and interview-reported trauma scores was sufficiently high for individual items, subscales, and TADS totals with no indication of a general bias towards either an under- or over-reporting. There was, however some indication of better inter-method reliability for raw score based subscale and TADS totals compared to dichotomised scores (those having any one included item with a frequency of at least “sometimes”). While the ICCs of all raw score sums indicated excellent agreement, κ values of dichotomised domains and totals were poorer and fell below the threshold for clinical utility for totals.

Inter-method reliability was poor overall for three items (2, 17, and 36), two of which had originally been part of the physical neglect and abuse domains, respectively, and negatively affected their internal consistency. These were removed from the respective domains and in the case of PhyAbR, replaced by an item with excellent inter-method reliability. A further five items (23, 31, 34, 38, and 39) possessed better inter-method reliability for their raw scores compared with dichotomised scores. Finally, EmoNeg appeared to be over-reported by self-report compared to interview, hence self-reports should be treated with some caution for this scale.

### Concurrent validity and normative data

Childhood adversity has frequently been associated with adult mental disorder, particularly depression (e.g., Fryers & Brugha, 2013; Kessler et al., 2010; Lindert et al., 2014), and so the DEPS screen positive cases and help-seeking for mental problems were used as proxy measures of the construct “traumatisation” in examining the TADS's concurrent validity and generation of norms. Effect sizes indicated small to moderate associations between proxy measures and TADS categories (caseness) that appeared to be quite robust. Because the relationship between childhood trauma and adult mental ill health is complex and significantly mediated by many interacting factors (Fryers & Brugha, 2013; Kessler et al., 2010), the small-to-moderate effect sizes suggest good concurrent validity of the TADS.

Using the same two proxies of clinically significant prior adversity as markers, TADS trauma domains and totals were assessed for their diagnostic relevance. We additionally examined the influence of age, gender and education to see if we could improve the population fit using different demographic norms (Michel et al., 2014). Education particularly seemed to relate to TADS scores and the inverse association appears supportive of research linking childhood adversities to impaired physical brain development (Bick & Nelson, 2016) as well as the impact on education (Font & Maguire-Jack, 2016) and studies linking education to poly-victimisation (e.g., Barker, Kerr, Dong, Wood & DeBeck, 2015; Horan & Widom, 2015; Min, Farkas, Minnes, & Singer, 2007).

Depression and help-seeking status also enabled an approximate comparison of diagnostic accuracy measures for TADS domain and total caseness. As expected, the total TADS (43 items) scale had very low specificity for proxy measures and therefore may not be suitable for detecting early traumatisation. Specifically for depressiveness, total TADS domains also demonstrated low specificity but higher specificity for help-seeking which is likely to be an indicator of a much wider range of psychiatric symptoms or disorders and thus indicates the instrument's clinical utility. Because of the low reported frequency of sexual abuse events, sensitivity for SexAb remained low, but its high specificity and moderate positive LR for depressiveness support the view that childhood sexual abuse is specifically related to clinical depression in adulthood (Lindert et al., 2014). However, with regard to specificity, positive predictive value (PPV) and LRs in particular, the limited nature of depression and help-seeking as proxy measures of “traumatisation” in a general population sample has to be kept in mind.

### Strengths and limitations

In addition to the good psychometric properties of the TADS indicated by the present results, some further strengths as well as limitations should be discussed. While the TADS data presented is from a large adult general population and primary care samples with broad age ranges, the high level of non-responders may limit the representativeness of results and might have biased the reporting of childhood adversity, depression, and help-seeking. Females and young adults were particularly over-represented among subjects. In addition, it must be noted that the Finnish population is very demographically homogenous (97% spoke the official native language Finnish/Swedish) and the proportion of non-Caucasian people is very low (under 1%). This fact clearly limits generalisation of the results to other countries with more multicultural populations.

Altogether 95% of participants affirmed at least one TADS item as having occurred “sometimes” or more often. Most frequent were reports of childhood and family “being perfect” and “the greatest ever,” respectively, of doing well at school, having trusted friends, and of having experienced the loss of an important person. None of these items are part of the five core domains, and, consequently, when only 24 domain items were considered, the hit rate reduced considerably. Figures for emotional (51.2%) and physical (49.7%) neglect and for emotional (37.4%) and physical (23.1%) abuse were considerably higher than in some other studies (Barbosa et al., 2014; Christoffersen, Armour, Lasgaard, Andersen, & Elklit, 2013; Kessler et al., 2010; Saed et al., 2013; Schüssler-Fiorenza Rose et al., 2014), while prevalence of SexAb (5.5%) was as frequent as in German (Iffland et al., 2013) and Brazilian population samples (Barbosa et al., 2014) but lower than in the Boston area study (Chiu et al., 2013) and higher than in the WHO study (Kessler et al., 2010). However, the use of different instruments and definitions of adversity impede direct comparisons between separate studies (Burgermeister, 2007; Thabrew et al., 2012). For example in the ACE study (Schüssler-Fiorenza Rose et al., 2014), the emotional abuse category included only one item with a description of adverse events (prevalence: 34%), while the TADS, for which the five emotional abuse items included also milder events, reported 51% prevalence. It is also possible that recent public and media discussions on childhood adverse experiences in Finnish society have increased reporting for milder adverse events or experiences. While the high reporting of prior adversity reported by this sample may indicate a questionnaire return bias, the rates of reported depression scores according to the DEPS of 20% and that of lifetime help-seeking of 27% is in line with the previous prevalence reports of mild-to-moderate self-reported depression symptoms of 14% in adults of the Finnish community sample (Koivumaa-Honkanen, Kaprio, Honkanen, Viinamäki, & Koskenvuo, 2004) and of help-seeking for mental health problems of 23% in young adults of a Swiss community sample (Schultze-Lutter, Michel, Ruhrmann, & Schimmelmann 2014). Thus, it is unlikely that a return bias towards more distressed individuals has driven the high reported rate of childhood adversity.

One limitation that is inherent to the construct of childhood adversity and trauma and consequently relates to all similar studies is the lack of a “gold standard” measure for the retrospective assessment of the complex construct “traumatisation.” Thus, when comparing the concurrent validity of adversity and trauma assessments, much depends on the quality of proxy measures of the construct. Based on consistent reports of a causal link between childhood adversities, traumatisation, and adult mental ill health (e.g., Fryers & Brugha, 2013; Kessler et al., 2010; Lindert et al., 2014), we had chosen a self-report measure of current depression and report of lifetime help-seeking for mental disorders that despite their differing time frames of reference led to impressively similar results. The proxy measures were limited in that only current depressiveness was assessed thus potentially excluding any earlier depressive episodes and help-seeking only from psychiatric services was assessed, yet help-seeking can involve other providers such as primary health services or indeed help might not be sought at all (Kaskeala, Sillanmäki, & Sourander, 2015). Future evaluations of the TADS might usefully employ measures of hypothalamic–pituitary–adrenal (HPA) axis dysregulation as a neurobiological marker and proxy measure of childhood traumatisation. The HPA axis is a major part of the neuroendocrine system that controls reactions to stress, involved in the neurobiology of many mental disorders (Baumeister, Lightman, & Pariante, 2014) and permanently modulated by early life stressors (Macrì, Zoratto, & Laviola, 2011). While exposure to mild or moderate stressors early in life has been shown to enhance HPA regulation and promote a lifelong resilience to stress, early-life exposure to extreme or prolonged stressors can induce a hyper- or hypo-reactive HPA axis and may contribute to lifelong vulnerability to stress (Flinn, Nepomnaschy, Muehlenbein, & Ponzi, 2011; Hinkelmann et al., 2013). Similarly, future studies of the concurrent validity of assessments of childhood trauma could also consider employing cortisol—in particular hair cortisol that reflects cumulative cortisol levels over long periods of time—as a measure of potential HPA axis dysfunction and thus a neurobiological proxy of traumatisation (Hostinar & Gunnar, 2013).

### Conclusions and outlook

In relation to measuring the important role that early-life traumatisation plays in the development of adult mental health problems and disorders, many instruments have been developed based on face validity, with relatively few reporting psychometric properties (Burgermeister, 2007; Pietrini et al., 2010; Thabrew et al., 2012). Regarding the TADS and its five revised sub-scale domains, our results indicate good psychometric properties in terms of internal consistency, content, inter-method reliability, and concurrent validity for adults from a Finnish community sample. These findings require replication and our suggested cutoff markers for clinical significance and traumatisation, respectively, will need validation with independent samples such as clinical populations or in other regions employing different proxy measures of traumatisation (including neurobiological) and prospective studies. In addition, the test–retest reliability of the TADS and its applicability to younger samples should be reported. As regards the TADS's utility, it seems possible to improve this while retaining good content validity in terms of the five core domains of childhood trauma by employing only 24 of the measures items.

Overall, the TADS appears to be a useful instrument for the assessment of retrospectively reported childhood adversity and trauma beyond the contextual framework of its original development for the prediction of psychosis in clinical high-risk samples.

## Supplementary Material

Psychometric properties of the Trauma and Distress Scale, TADS, in an adult community sample in Finland
