# Supplementary material for: Psychometric properties of the Trauma and Distress Scale, TADS, in an adult community sample in Finland
Source: Eur J Psychotraumatol. 2016 Mar 30;7:10.3402/ejpt.v7.30062. doi: 10.3402/ejpt.v7.30062 (PMC4816812; doi:10.3402/ejpt.v7.30062)
Supplement: Psychometric properties of the Trauma and Distress Scale, TADS, in an adult community sample in Finland [file EJPT-7-30062-s001.pdf]

Appendix

The traumatic and distress experiences scale

TADS – *EPOS* version 1.2

<u>Instructions</u>

These questions ask about personal experiences you may have had in your life so far.

Many questions refer to *'when you were young':* this means the period of your life when you were growing up and before you left school. When we talk about *'parents'* this means the adults who had the main responsibility for your upbringing as a child and teenager.

If your parents behaved differently, please answer the questions thinking about the parent whose behaviour was worse.

Read each item carefully and tick the box that most accurately describes the experience from your point of view. Please answer all the questions as honestly as you can.

Thank you for your time

© Patterson, Skeate, Schultze-Lutter, von Reventlow & Wienecke 2002

	Never	Rarely	Some-times	Often	Nearly Always
1. When I was young, I felt safe and protected by somebody.					
2. When I was young, I was often hungry.					
3. I was bullied at school.					
4. I often had to wear ragged or dirty clothes to school.					
5. When I was young, I felt valued or important.					
6. My parents / caregivers were often drunk, stoned or wasted.					
7. I have been bullied at work.					
8. My family were emotionally warm and loving.					
9. When I was young, I was hit so hard that it left marks, cuts or bruises.					
10. I felt rejected by my parents / caregivers.					
11. When I was young, there was an adult I could confide in.					
12. When I was young, I was humiliated by people in my family.					
13. When I was young, my family looked after each other.					
14. I believe that I am a bad person.					
15. I believe that somebody died because of me.					
16. I have experienced serious physical assault.					
17. Adults (like teachers, doctors or nurses) noticed cuts, bruises or marks from when I was beaten.					
18. My childhood was perfect.					
19. I am bothered by a very shameful secret.					
20. I think I was physically abused when I was young.					
21. I respect myself.					
22. When I was young, someone touched me or tried to make me touch them in a sexual way.					
23. I have had experiences that I feel very guilty about.					
24. I have been involved in life-threatening situations.					
25. I was forced to keep secrets about someone sexually interfering with me when I was young.					
26. When I was young, I felt hated by a member or members of my family.					
27. My family was the greatest ever.					
28. Other people have acted badly because of me.					
29. When I was young, I felt like the odd one out in my family.					
30. I have experienced sexual assault.					
31. If I needed treatment someone would always take me to see a doctor or nurse when I was young.					
32. I feel that I was put down, criticized and made to feel inferior when I was young.					
33. Someone sexually molested me when I was young.					
34. I feel responsible for harm or injury to another person.					
35. When I was young, I had friends I could talk to about personal problems.					
36. I have experienced harassment / persecution from other ethnic groups.					
37. I did well at school.					
38. I have experienced the loss of somebody who was very important to me.					
39. I believe that I do not deserve to do well in life.					
40. My family was supportive and encouraging when I was young.					
41. I believe that I was sexually used when I was young.					
42. I felt afraid of someone in my family.					
43. When I was young I could make friends easily					
